# The effects of calcitriol on improvement of insulin resistance, ovulation and comparison with metformin therapy in PCOS patients: a randomized placebo- controlled clinical trial 

**Published:** 2012-09

**Authors:** Shokoufeh Bonakdaran, Zahra Mazloom Khorasani, Behrooz Davachi, Javad Mazloom Khorasani

**Affiliations:** 1 *Endocrine Research Center, Mashhad University of Medical Sciences, Mashhad, Iran.*; 2 *Department of Radiology, Radiology Center, Mashhad University of Medical Sciences, Mashhad, Iran.*; 3 *Imam Reza Hospital, Mashhad, Iran.*

**Keywords:** *PCOS*, *Ovulation*, *Vitamin D*, *Insulin resistance*

## Abstract

**Background:** Polycystic ovary syndrome (PCOS) is the most common endocrine disorder in females of reproductive age. Insulin resistance is a frequent metabolic disturbance in PCOS. Vitamin D deficiency is a common problem. Accumulating evidence suggests that vitamin D has a role on insulin sensitivity so may contribute to reduction of hyperandrogenemia.

**Objective:** The aim was to determine the effects of vitamin D treatment in metabolic components and ovulation evidence in PCOS.

**Materials and Methods:** Fifty one untreated PCOS patients were randomly divided into three groups and treated with calcitriol, metformin, or placebo. Before and 3 months after treatment, ovulation evidence was assessed by ovarian trans abdominal sonography. Plasma fasting glucose, insulin, homeostasis model assessment insulin resistance (HOMA-IR), 25-hydroxyvitamin D, parathyroid hormone and androgen levels were measured before and after treatment. A 75gr glucose test was performed before and after treatment and two set of results was compared.

**Results:** Three patients did not continue this study. Only 11 patient (22.9%) had sufficient vitamin D levels (>30 ng/ml). Metformin caused a significant decrease in weight (p=0.027), insulin level (p=0.043), and insulin resistance (p=0.048). Systolic blood pressure and PTH significantly improved after calcitriol (p=0.029, p=0.009 respectively). An improvement in ovulation was detected after calcitriol and seven patients, without evidence of ovulation before treatment, illustrated ovulation after 3 months. Difference with calcitriol in ovulation was significant versus other two methods (p=0.02).

**Conclusion:** Calcitriol treatment in PCOS may be prior to metformin in ovulation induction.

## Introduction

Polycystic ovary syndrome (PCOS) with a prevalence of about 5-10% in different communities is the most common endocrine disease in reproductive age women (1). This syndrome is diagnosed with two criteria of three: An unovulation or oligoovulation, clinical or laboratory markers of increase in androgen levels and diagram of PCO in sonography (2). 

Insulin resistance and increase in insulin levels resulted from resistance to it and also other components of metabolic syndrome including: glucose intolerance, lipid disorder and increase in blood pressure is common in PCOS (3). New researches are confirmed the probable role of vitamin D in secretion of insulin and improvement of insulin resistance and even it seems that deficiency of vitamin D is probable factor in metabolic syndrome pathogenesis (4, 5). Some of studies show that treatment with vitamin D has useful effect on decrease in insulin resistance, blood sugar, lipid profiles and blood pressure and even decrease in body weight in diabetic patients or patients with metabolic syndrome (6, 7). There are similar limited studies at the same topics about patients with PCOS. 

Hahn *et al* in 120 untreated PCOS patients demonstrated that women with hypovitaminosis D had higher BMI and insulin resistance (8). Regarding to interference of insulin resistance in increasing of androgen level and then atresia of growing follicle in patients with PCOS, it seems that treatment with vitamin D can be effective in metabolic improvement, reduction in androgen levels, and even improvement of ovulation in patients with PCOS. 

The effects of vitamin D treatment in parameters of glucose metabolism in PCOS patients examined by Kotsa *et al* They concluded that treatment with the vitamin D analogue (alphacalcidol) could be of value in augmentation of first phase of insulin secretion and improvement of lipid profiles (9). A randomized clinical trial that conducted by Rashidi *et al* in 60 infertile PCOS patients showed that the number of dominant follicles (≥14mm) during the 2-3 months of treatment was higher in the calcium-vitamin D plus metformin group than in only metformin or only calcium-vitamin D treatment (p=0.03) (10). The role of metformin has been proven as one of common treatment of PCOS in improvement of metabolic complications and ovulation in these patients (11, 12). 

In this study, we tried to compare therapeutic effects of metformin and calcitriol (active form of vitamin D) in improvement of metabolic factors, clinical evidence and sonographic changes, which prove ovulation in patients with PCOS.

## Materials and methods

This study was designed for clinical trial which has coincided usage of control group under treatment of placebo. Fifty-one women which had diagnostic criteria of PCOS were selected out of patients who have outpatient refer to clinics of Ghaem hospital because of common complaint of this syndrome. Number of Sample size selected according to the Kotsa *et al* study on the basis of reduction of insulin resistance after vitamin D treatment. Diagnosis criteria of these patients were based on Rotterdam diagnosis criteria for PCOS. 

Two criteria of these three were necessary: 1) unovulation or oligoovulation (or menstrual cycle less than 6 cycles in 12 month) 2) Clinical or biochemical findings of hyperandrogenism including: acne, hirsutism, alopecia or increased serum androgen levels. 3) View of polycystic ovary with 12 follicles or more in each ovary with diameter between 2-9 mm and ovarian volume increasing more than 10 cm^3^. 

For diagnosis of PCOS, other cause of hyperandrogenism should be rule out including: congenital adrenal hyperplasia, Cushing's syndrome, hyperprolactinemia, hypothyroidism and tumors that secrete androgens were excluded by laboratory markers including 17 α hydroxy progesterone, ACTH test in suspicious cases, prolactin, cortisol level after overnight dexamethasone and thyroid test. Also regarding to check of vitamin D level in patients, all systemic interfering causes on level of this vitamin, like renal disease, hepatic disease, malabsorption, alcoholism, breast feeding, pregnancy and all users of drugs which were effective in level of vitamin D, like anti-epileptic drugs, calcium and vitamin complement, glucocorticoid, were excluded from this study. 

After selection of patients the questionnaires by all the patients was filled including height, weight in standard situation, measurement of blood pressure, score of hirsutism, menstrual cycle status and existence or absence of infertility. Laboratory analysis was performed in follicular phase if menstrual cycle present and otherwise in the 2-6 day of the menstrual cycle after use of progesterone after 8 hours fasting. 5cc Venous blood sample was drown for measurement of FBS, lipid profile, calcium, phosphor, insulin, testosterone level, PTH, DHEAS, and 25 hydroxy vitamin D. 

Blood Sugar was checked with glucose oxidase method. Lipid, calcium, and phosphor were measured with enzymatic method and insulin with immunoradiometric method (immunotech kit, Beckman Company) with intra-assay CV 3.4% and inter-assay CV 4.3%. 

Testosterone was determined using radioimmunoassay ( intra-assay CV 15% and inter-assay CV 14.8%) and DHEAS with radioimmunoassay method with inter-assay CV 10.8% and intra-assay 7.4% and vitamin D was measured by radioimmunoassay method (bio source kit) with intra-assay CV 5.2% and interassay CV 7.5%. Then all the patients underwent a glucose tolerance test with 75 gr glucose and glucose level was checked after 120 minutes after ingestion of glucose. Insulin resistance was calculated according to the formula: HOMA-IR= FBS (mmol/lit) × fasting insulin (microunit/mlit) /22.5. 

Ovarian transabdominal sonography was done for all the patients if there is any cycle in the mid menstrual cycle and otherwise after the cycle following progesterone and evidence of ovulation was checked on the basis of presence or absence of dominant follicle more than 12 mm. 

The patients were then randomized into three groups based on days of the week on which they referred to clinic; Group I: patients referred on Saturday and Sunday, Group II: patients referred on Monday and Tuesday and group III: who referred on Wednesday and Thursday. After randomization, Group 1 treated by 1000 mg/day metformin (metformin Hexal) for three months. In Groups 2 patients treated by 0.5 microgram/day calcitriol (Zahravi, Tabriz, Iran) for 3 month. 

The reason for the selection of this compound was because of short half life and also reassurement of therapeutic effects on people who have reduced the vitamin D activation and Groups 3 underwent placebo treatment in the form of synthetic drug like metformin, during 3 months. During this 3 month period, patients referred if they would have side effect. 

Two patients in group 2 and one patient in group 3 discontinued treatment and they excluded from study. Other patients came again and all the primary tests were requested for all the patients. Second questioner was filled for the changes in weight, blood pressure, menstrual cycle for all patients. Ovarian sonography was done again for patients and checks the changing favor to ovulation. 

**Statistical analysis**

Statistical analysis was done with SPSS 11.5. Data was expressed as mean±SD. ANOVA test was used for analysis of normal distribution variable and Kruskal Wallis for non-normal distribution variable. Paired t-test was used for normally distributed variables and Wilcox on for comparison of not normally distributed data before and after treatment. Categorical variables were compared by crosstab test. The relationship between variables was interpreted with Pearson in normal distribution and with Spearman in non-normal distribution. P<0.05 was considered significant in all studies. All patients gave informed voluntary consent and the study protocol was approved by the research ethics committee of Mashhad University of medical sciences.

## Results

**Figure 1:**
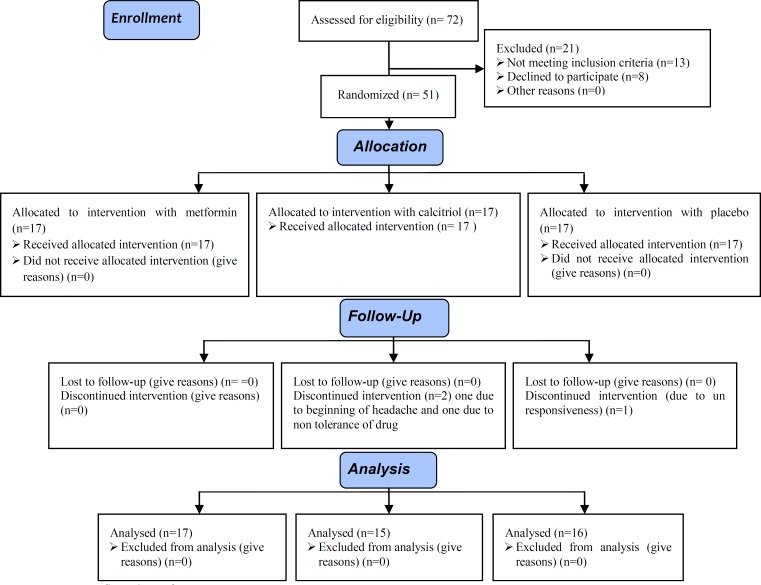
Consort flow chart of RCT.

Among 51 selected patients, 3 people didn’t continue study. One person was on placebo groups and two other were in calcitriol groups. So analysis was done on 48 remaining patients (Figure 1). Table I shows primary demographic, clinical and laboratory characteristics in 3 groups of patients and their statistical significance. 43 patients had hirsutism. 

Which in 21 of them, hirsutism score was less than 8, 14 patients had hirsutism score 8-12 and 8 patients had severe hirsutism with score more than 12. In first sonography before treatment 37 patients (77.1%) were without ovulation evidence and 11 patients (22.9%) had ovulation evidence. In this study only 11 patients (22.9%) had sufficient 25 hydroxy vitamin D levels (>30 ng/ml). 

Patients randomly divided in 3 therapeutic groups (metformin, calcitriol, and placebo) and after three month analysis was done again. Laboratory results comparison before and after treatment in 3 groups is illustrated in table II. As is clear from the results of this table, treatment with metformin significantly decreased insulin level and insulin resistance that this metabolic effect was not seen in other groups. Treatment with calcitriol caused an improvement in 25 hydroxy vitamin D level and significant reduction in PTH level that this effect were not seen in other groups.

To compare the response of follicle for improvement of ovulation (size of dominant follicle) following interventional treatment Cross tab test was used that result of it is shown in table III that as it is clear, treatment with calcitriol significantly improved ovulation in patients. We compared ovulation evidence in each groups before and after interventional treatment by sonography. 

In interventional treatment groups with metformin and placebo this changes were not obvious. (p=0.42, 0.67 respectively) but treatment with calcitriol improved ovulation significantly (p=0.003) and even two infertile patients treated by calcitriol after 2-3 month cut in treatment with calcitriol experience natural gestation that this effect were not seen in metformin and placebo treatment. 

In numeral correlation analysis between laboratory variables a significant reverse correlation was found between 25 (OH) D serum level and level of DHEAS (p=0.01, r=-0.37). Relationship between levels of 25 (OH) D and other laboratory variables and also weight and blood pressure were not significant.

**Table I:** Baseline clinical and biochemical variables of the PCOS patients

**Variable**	**Metformin treatment ** **(17 patients)**	**Calcitriol treatment ** **(15 patients)**	**Placebo treatment ** **(16 patients)**	**p-value**
Age (years)	25.9 4.5	24.7 3.3	25.2 7.9	0.84
Body mass index (BMI)	28.2 5.03	24.8 5.3	25.3 5.1	0.14
Systolic blood pressure (mm hg)	113.3 12.9	110.0 6.7	107.6 14.8	0.39
Diastolic blood pressure (mm hg)	72.6 7.03	70.7 6.1	71.5 8.9	0.69
Fasting blood sugar (FBS) (mg/dl)	99.7 29.7	81.7 8.6	86.3 5.4	0.08
Blood Sugar (BS) 2h after glucose (mg/dl)	118.7 60.1	92.2 21.4	92.9 18.8	0.11
Insulin (micU/mlit)	29.9 30.8	18.3 30.4	13.6 14.6	0.19
HOMA-IR	8.8 12.8	4.2 6.8	2.8 2.9	0.19
Calcium (mg/dl)	9.3 0.4	9.4 0.4	9.5 0.4	0.48
Phosphorous (mg/dl)	4.0 1.0	3.9 0.7	4.0 0.6	0.77
Cholesterol (mg/dl)	170.7 19.9	164.0 31.3	173.0 35.3	0.35
LDL (mg/dl)	94.1 19.4	101.6 21.6	107.3 29.5	0.79
HDL (mg/dl)	44.5 8.4	46.5 10.9	45.3 7.9	0.99
Triglyceride (mg/dl)	173.5 165	88.5 38.3	99.7 48.2	0.01
Testosterone ( ng/dl)	88.2 48.1	94.5 49.4	88.0 43.7	0.71
DHEAS (ng/ml)	2643.7 1530.8	2649.7 1341.4	3168.6 1642.1	0.36
25 (OH) D (ng/ml)	28.2 13.5	11.4 8.2	19.9 16.5	0.01

HOMA-IR, homeostasis model assessment as an index of insulin resistance; LDL, low density lipoprotein; HDL, High density lipoprotein; DHEAS, Dehydroepiandrosterone sulfate.

Data are expressed as means±SD.

P<0.05 is considered significant.

ANOVA test was used for analysis of normal distribution variable and Kruskal Wallis for non-normal distribution variable.

**Table II:** Comparison of parameters in PCOS patients before and after treatment with metformin, calcitriol and placebo

**Variable**	**Metformin treatment**			**Calcitriol treatment**			**Placebo treatment**		
	**Before**	**After**	**p-value**	**Before**	**After**	**p-value**	**Before**	**After**	**p-value**
Weight (kg)	74.613.7	72.914.4	0.02	63.014.2	62.513.9	0.54	63.714.7	63.014.8	0.23
Systolic blood pressure (mmhg)	113.312.9	106.614.9	0.07	110.06.7	105.09.4	0.02	107.614.8	109.213.2	0.54
Diastolic blood pressure (mmhg)	72.67.03	70.610.3	0.38	70.76.1	69.26.1	0.50	71.58.9	72.38.3	0.67
FBS (mg/dl)	99.729.7	102.025.5	0.54	81.78.6	89.012.3	0.14	86.35.4	87.35.3	0.62
BS 2 hour after glucose (mg/dl)	118.760.1	114.042.5	0.68	92.221.4	95.013.8	0.70	92.918.8	93.619.1	0.90
Insulin (micU/lit)	29.930.8	14.215.1	0.04	18.330.4	13.114.8	0.40	13.614.6	8.65.0	0.31
HOMA-IR	8.812.8	4.26.9	0.04	4.26.8	2.73.1	0.36	2.82.9	1.91.0	0.38
Calcium (mg/dl)	9.30.4	9.40.3	0.48	9.40.4	9.40.4	0.95	9.50.4	9.60.9	0.73
Phosphorous (mg/dl)	4.01.0	3.80.4	0.72	3.90.7	3.90.6	0.76	4.00.6	3.60.8	0.20
PTH (pg/ml)	38.318.5	64.223.5	0.01	60.325.9	31.422.8	0.009	45.421.9	43.620.4	0.79
Testosterone (ng/dl)	88.248.1	107.162.0	0.27	94.549.4	106.631.7	0.51	88.043.7	122.565.0	0.18
DHEAS (ng/ml)	2643.71530.8	2816.21684.1	0.40	2649.71341.4	2645.01071.2	0.98	3168.61642.1	3335.81546.5	0.56
25 (OH) D (ng/ml)	28.213.5	26.710.6	0.48	11.48.2	20.116.2	0.06	19.916.5	19.015.3	0.74

FBS, fasting blood sugar; HOMA-IR, homeostasis model assessment as an index of insulin resistance; LDL, low density lipoprotein; HDL, high density lipoprotein ; DHEAS, Dehydroepiandrosterone sulfate .

Data are expressed as means±SD.

P-value<0.05 is considered significant.

Paired t test was used for normally distributed variables and Wilcoxon for comparison of not normally distributed data before and after treatment.

**Table III:** Comparison of ovulation findings in PCOS patients before and after treatment with metformin, calcitriol and placebo

**Ovulation findings**		**Metformin group** **(17 patients)**	**Calcitriol group** **(15 patients)**	**Placebo group** **(16 patients)**	**p-value**
First sonography before treatment					
	Without	15 (88.2%)	11 (73.3%)	11 (64.7%)	0.23
	With	2 (11.7%)	4 (26.7%)	5 (35.5%)	
Second sonography after treatment					
	Without	13 (76.4%)	4 (26.7%)	9 (56.3%)	0.02
	With	4 (23.6%)	11 (73.7%)	7 (43.7%)	

Data are expressed as number (%).

P-value<0.05 is considered significant.

Categorical variables were compared by crosstab test (Chi square).

## Discussion

Vitamin D deficiency in patients with PCOS was very obvious in this study. Only 22.9% of our patients had sufficient vitamin D level that this result is comparable whit some other results published in this area. Wehre *et al* studies that Hypovitaminous D in 72.8% of PCOS patients that this result is very similar to our current study (13). In some studies vitamin D deficiency in obese PCOS patients was more than patients with normal weight and these studies has been shown a reverse significant relationship between vitamin D level and body mass index (BMI). It seems that reverse relationship between vitamin D and obesity is bilateral. On the one hand obesity cause a decrease in vitamin D flow with trapping of vitamin D in adipose tissue (14) and in other hand looks that obese person due to physical inactivity have less fully benefit from sun exposure that constituent in vitamin D synthesis (15). Lack of contact with sun in patients with PCOS with sever hirsutism is more due to shaming of social participation. In our study relationship between 25 hydroxy vitamin D level and BMI were not significant (p=0.94) and also patients treated with calcitriol not shows a significant change in weight and BMI after 3 months.

In frequent studies has been pointed to the role of deficiency of vitamin D in augmentation of insulin resistance, impaired glucose tolerance test and progression to diabetes. It seems that vitamin D has an important role on insulin secretion from pancreas beta cells and also in sensitivity to insulin with stimulation of insulin’s receptors. In some of studies on PCOS patients has seen a reverse relationship between vitamin D levels and insulin resistance (16) that some part of this relationship maybe related to coincident obesity in these patients. 

Role of vitamin D is important in modulation of immune system and vitamin D deficiency accompanies with more inflammatory responses that it can be a reason for increasing in insulin resistance. In our study a significant relationship between vitamin D level with insulin level and insulin resistance was not seen. Although after receiving calcitriol, insulin level and HOMA-IR has decreased but this rate of changes was not statistically significant. 

Reduction in insulin level and insulin resistance in metformin groups was significant after treatment. In a lot of studies pointed relationship between vitamin D deficiency and progression of diabetes and metabolic syndrome and even some of studies shows that use of vitamin D supplements cause an improvement in component of metabolic syndrome in patients. In our study has not been seen significant relationship between vitamin D level and fasting blood sugar (p=0.14), blood glucose two hours after 75 gr glucose (p=0.88) and even treatment with vitamin D cause an trivial increase in fasting sugar that this change was not significant.

Several explanations for difference our report with other studies in metabolic aspect is possible. This study was short in duration and included few subjects. Racial differences and its influence on VDR polymorphism and also difference in type of vitamin D compound, dosage and duration of drug usage are probable factors for difference of our results with other studies. But it should be reminded that there are a lot of controversies due to improvement of metabolic data and also in other performed studies, and there is no compromise (17, 18).

One of the most important results of this study was a significant impact of calcitriol treatment on ovulation. High percentage of patients that has not ovulation evidence in the first sonography, after interventional treatment showed that evidence and even two patients experienced natural pregnancy. Role of calcium in oocytes and follicle maturation was proven so many studies (19). Even in a study it is shown that adding calcium ion into culture field improves the halt maturation of oocyte in meiosis due to reduction of intracellular calcium (20). Also it seems that vitamin D promote apoptosis property and vitamin D deficiency result decrease in apoptosis and increase in anti-apoptosis factor that may be a factor to creation of PCOS and disruption of normal ovarian folliculogenesis (21). Although in our study calcium level in all patients was normal but measurement of serum calcium is not a good marker for estimation of intra cellular calcium. 

Furthermore, looking at the rate of vitamin D deficiency in our study, it was clear that maybe this deficiency independently of calcium cause disturbance on natural progress of ovarian follicles. Effect of vitamin D components on improvement of ovulation in PCOS patients reported in two other studies (18, 19). Another important result of this study was a reverse significant relationship between vitamin D level and a level of DHEAS. Part of this effect may be related to role of vitamin D in insulin resistance and effect of insulin resistance on increase androgens levels that in our study had not seen significant relationship between vitamin D level and insulin resistance. 

So probably this relationship is an independent relationship from effect of insulin resistance. It seems that vitamin D can stimulate aromatase activity that effective in conversion of testosterone to estrogens in granulosa cells that this matter cause a balance on androgen and estrogens level in patients with PCOS (22). Although being of this reverse relationship between vitamin D and DHEAS pointed in one another study but we couldn’t find cause of this relationship. Finally regarding to prevalence of vitamin D deficiency in our country ,that pointed to in so many studies and probable role of vitamin D in ovulation improvement, easy and inexpensive access to this drug it seems that complementary use of this drug will be useful as well as the other recommended drugs for PCOS.

## Footnote

***Registration ID in IRCT: IRCT201108074167N4.***
